# Protective Effects of Ellagitannin-Rich Strawberry Extracts on Biochemical and Metabolic Disturbances in Rats Fed a Diet High in Fructose

**DOI:** 10.3390/nu10040445

**Published:** 2018-04-04

**Authors:** Bartosz Fotschki, Jerzy Juśkiewicz, Krzysztof Kołodziejczyk, Adam Jurgoński, Monika Kosmala, Joanna Milala, Katarzyna Ognik, Zenon Zduńczyk

**Affiliations:** 1 Institute of Animal Reproduction and Food Research, Division of Food Science, Tuwima 10, 10-748 Olsztyn, Poland; a.jurgonski@pan.olsztyn.pl (A.J.); z.zdunczyk@pan.olsztyn.pl (Z.Z.); 2 Institute of Food Technology and Analysis, Lodz University of Technology, Stefanowskiego 4/10, 90-924 Lodz, Poland; krzysztof.kolodziejczyk@p.lodz.pl (K.K.); monika.kosmala@p.lodz.pl (M.K.); joanna.milala@p.lodz.pl (J.M.); 3 Department of Biochemistry and Toxicology, Faculty of Biology, Animal Sciences and Bioeconomy, University of Life Sciences, 20-950 Lublin, Poland; kasiaognik@poczta.fm

**Keywords:** monomeric ellagitannins, nasutin A, microbiota activity, glutathione, inflammatory cytokines

## Abstract

The present study compares the effects of two dietary strawberry extracts rich in monomeric (ME) or dimeric (DE) ellagitannins (ETs) on gastrointestinal, blood and tissue biomarkers in Wistar rats fed high-fructose diets. Both strawberry extracts beneficially affect the antioxidant status and lipid profile of the liver and serum. The ME extract shows a greater ability to inhibit lipid peroxidation in kidneys, more effectively decreases serum and liver triglycerides, and exerts greater anti-inflammatory effects in blood serum than the DE extract. The DE extract significantly reduces the activity of microbial enzymes in the cecum. These effects might be associated with higher cecum and urine levels of ET metabolites in rats fed with ME than in rats fed with DE. In conclusion, the diet-induced fructose-related disturbances observed in biochemical parameters are regulated by both extracts; nevertheless, the beneficial effects of the ME extract are mostly associated with systemic parameters, while those of the DE extracts are associated with local microbial activity.

## 1. Introduction

Many studies have supported the claim that a diet enriched with berries contributes to health benefits in consumers worldwide [1,2,3]. Similar to other berries, strawberries (*Fragaria* × *ananassa*) contain imposing levels of bioactive compounds such as essential vitamins, minerals, fatty acids, dietary fiber, and polyphenols [4]. Among the latter, ellagitannins (ETs), anthocyanins, flavonols and flavanols are the major strawberry phytochemicals with antioxidant, anti-mutagenic, antimicrobial and anti-inflammatory properties [3,5,6]. It has been reported that dietary ETs should be considered very promising tools for multi-targeted therapy of several diseases [7,8,9].

To the best of our knowledge, there are only a few reports focused on the physiological response of laboratory animals fed dietary ETs with different degrees of polymerization. It is well known that even a small change in the chemical structure of a molecule is of paramount importance to its biological activity in the host’s body [10,11,12]. Our recent study showed that the separate mechanisms leading to decreased postprandial glycaemia upon dietary administration of strawberry ETs largely depends on their chemical structure, i.e., degree of polymerization [13]. In the study on rats it was presented that extract with monomeric ETs was mitigated the glucose- or sucrose-induced postprandial glycemic load more beneficially than extract with dimeric ETs [13]. Another study by Jurgoński et al. [14] showed strong antibacterial activity of a dietary combination of monomeric strawberry ETs with fructooligosaccharides (FOSs) in the rat cecum, and this effect was not observed with dimeric ETs or in treatments without FOSs. Kosmala et al. [15] in nutritional study on rats showed that lower content of less-polymerized ETs in the diet considerably elevated cecal concentration of ET metabolites. The concentration and profile of the ET metabolites is related with activity of the gut microbiota that can convert ETs to more bioavailable compounds and thus indirectly modulate systemic parameters [16].

Given the observed findings in our recent experiments regarding the effects of strawberry ETs with different degrees of polymerization on gastrointestinal and metabolic-related indices, while preparing the experimental schema of this study, we expect that monomeric and dimeric ET-rich dietary extracts would beneficially but, to some extent, differentially alleviate disturbances associated with high fructose administration. 

## 2. Materials and Methods

### 2.1. Preparation of Strawberry ET-Rich Extracts and Their Analysis

Extracts were obtained from strawberry fruit pomace, a by-product of the manufacture of concentrated strawberry juice (ALPEX Co., Łęczeszyce, Poland), as described by Jurgoński et al. [14]. In brief, the fresh pomace was dried in an industrial vacuum dryer at 70 ± 2 °C and then passed through sieves. The seedless fraction was subjected to a two-stage extraction with a 60% aqueous solution of acetone. Next, after partial removal of the solvent via distillation, the resultant solutions were transferred onto a column packed with a polymeric resin (Amberlite XAD 16, Sigma-Aldrich, Poznan, Poland). The sugars and other water-soluble compounds present in the solutions were eluted from the column with water. Then, monomeric and dimeric ET-rich fractions were desorbed with 10% and 40% aqueous solutions of ethanol, respectively, concentrated to ca. 15% of dry matter and lyophilized. The methods used to determine the composition of the monomeric and dimeric ET-rich extracts (details in Table 1) are described below.

The basic chemical composition of the extracts was determined according to the official methods of AOAC (2005) using the following procedures: 940.26 (dry substance and ash), 920.152 (protein), 930.09 (raw fat), and 985.29 (total dietary fiber).

The concentrations of ETs, ellagic acid, anthocyanins and flavonols were determined in the extracts after their dilution in methanol (1 mg/mL) using HPLC (Knauer Smartline system with a photodiode array detector, Berlin, Germany) coupled with a Gemini C18 column (110 Å, 250 × 4.60 mm; 5 μm, Phenomenex, Torrance, CA, USA). Phase A was 0.05% phosphoric acid in water, Phase B was 0.05% phosphoric acid in 80% acetonitrile, the flow rate was 1.25 mL/min, the sample volume was 20 μL, and the temperature was 35 °C. The gradient was as follows: stabilization for 5 min with 4% Phase B, followed by 4–15% B for 5–12.5 min, 15–40% B for 12.5–42.5 min, 40–50% B for 42.5–51.8 min, 50–55% B for 51.8–53.4 min and 4% B for 53.4–55 min. The following standards were used for the identification of the polyphenols: ellagic acid, flavonols (quercetin-3-*O*-glucoside, kaempferol-3-*O*-glucoside, quercetin, kaempferol, and tiliroside), pelargonidin-3-*O*-glucoside (all from Extrasynthese, Genay, France), *p*-coumaroic acid (Sigma-Aldrich, Poznan, Poland), and samples of ETs, specifically, hexahydroxydiphenoyl-d-glucose and agrimoniin, obtained by semi-preparative HPLC as described by Sójka et al. [17]. The absorbance was measured at 280 nm (*p*-coumaroic acid, tiliroside, hexahydroxydiphenoyl-d-glucose and agrimoniin), 360 nm (ellagic acid, quercetin, kaempferol and kaempferol glycosides) and 520 nm (anthocyanins).

The concentration of proanthocyanidins in the extracts was determined by the HPLC method after proanthocyanidin breakdown in an acidic environment with an excess of phloroglucinol, according to the method of Kennedy and Jones [18]. The obtained breakdown products were separated using a Knauer Smartline chromatograph (Berlin, Germany) equipped with a UV–Vis detector (PDA 280, Knauer, Berlin, Germany) and a fluorescence detector (Shimadzu RF-10Axl, Kyoto, Japan) and coupled with a Gemini C18 column (110 Å, 250 × 4.60 mm; 5 μm, Phenomenex, Torrance, CA, USA). The separation conditions were described by Kosmala et al. [15]. The identification was performed at 280 nm using a UV–Vis detector and the following standards: (−)-epicatechin, (+)-catechin, (−)-epigallocatechin and their respective phloroglucinol adducts. Quantification was conducted by peak areas registered by a fluorescence detector (excitation wavelength: 278 nm; emission wavelength: 360 nm). Standard curves of (−)-epicatechin and (+)-catechin for terminal units and (−)-epicatechin-phloroglucinol adduct for extender units were used to quantify the breakdown products.

### 2.2. In Vivo Experiment

The experiment was conducted on 48 male Wistar rats weighing 176 ± 1.251 g, which were randomly assigned to one of six groups of eight rats each. The animals were maintained individually in metabolic cages under a stable temperature (21–22 °C), a 12-h light:12-h dark cycle and a ventilation rate of 20 air changes per hour. The rats were used in compliance with the European Guidelines for the Care and Use of Laboratory Animals (EU Directive 2010/63/EU), and the animal protocol was approved by the local institutional animal care and use committee (Permission No. 32/2012; Olsztyn, Poland). For 6 weeks, the rats had free access to tap water and semi-purified diets, which were prepared and then stored at 4 °C in hermetic containers until the end of the experiment (details in Table S1). The diets were modifications of a casein diet for laboratory rodents recommended by the American Institute of Nutrition [19]. The corn starch (C), corn starch and monomeric ET-rich extract (C + ME), and corn starch and dimeric ET-rich extract (C + DE) groups received a diet based on corn starch, whereas fructose was the main carbohydrate in the control with fructose (F) diet and in the diets of the fructose and monomeric ET-rich extract (F + ME) and fructose and dimeric ET-rich extract (F + DE) groups. Treatment diets were modified with strawberry extracts (ME or DE) added at the expense of corn starch. All diets had equilibrated amounts of dietary protein, fiber, and polyphenols, if any. Regarding the polyphenols, however, they differed in terms of the content of monomeric and dimeric ETs and proanthocyanidins. The diets fed to the C + ME and F + ME groups had a higher total ET content with a monomer-to-dimer ratio of 96 to 4. The diets fed to the C + DE and F + DE groups had a lower total ET content with a monomer to dimer ratio of 40 to 60.

### 2.3. Sample Collection and Basic Analyses

Individual feed consumption and body weight (BW) gain of rats were determined. Rats were deprived of feed overnight (10–12 h) prior to anesthesia with sodium pentobarbital (50 mg/kg body weight). At the termination of the experiment, the rats were weighed and anesthetized as mentioned above. After a laparotomy, blood samples were collected from the caudal vein, and serum was prepared by solidification and low-speed centrifugation (350× *g*, 10 min, 4 °C). Serum samples were kept frozen at −70 °C until assayed. Selected intestinal segments (small intestine, cecum and colon) and internal organs (heart, kidneys and liver) were removed and weighed. Samples of the ileal, cecal and colonic digesta were collected, and the pH was immediately measured using a microelectrode and a pH/ION meter (model 301; Hanna Instruments, Vila do Conde, Portugal). After small intestine removal, mucosa from the second quarter of the intestine was collected by scraping with glass slides on an iced glass plate. After homogenization with four parts of a cold physiological saline (*v*/*w*) and centrifugation for 10 min (10,000× *g*, 4 °C), the obtained supernatant was stored at −40 °C until analysis. The mucosal disaccharidase activity (sucrase, maltase, and lactase) was assayed using a procedure described by Jurgoński et al. [20]. An aliquot of mucosal homogenate (0.1 mL) was incubated at 37 °C with 0.1 mL of a substrate solution (0.056 mol/L sucrose, maltose, or lactose in 0.2 mol/L phosphate buffer, pH 7.0). After 30 min of incubation, 0.8 mL of cold distilled water was added, and the enzymatic reaction was interrupted by immersion of the test tube in boiling water for 2 min. In addition, a blank with the same composition was prepared and immersed in boiling water without prior incubation at 37 °C. The released glucose was determined using a glucose oxidase reagent (Alpha Diagnostic, Ltd., Warsaw, Poland). The disaccharidase activity was expressed as µmol of glucose liberated from the respective disaccharide per min per g of protein. The mucosal protein content was estimated using the Bradford method, with bovine serum albumin as the standard.

In the fresh cecal digesta, the dry matter was determined at 105 °C, whereas the ammonia concentration was determined by the microdiffusion method in Conway’s dishes. After storage of the cecal digesta at −70 °C, the short-chain fatty acid (SCFA) concentrations were measured using gas chromatography (Shimadzu GC-2010, Kyoto, Japan) and a capillary column (SGE BP21, 30 m × 0.53 mm; SGE Europe Ltd., Milton Keynes, UK), as previously described [15]. The concentrations of cecal putrefactive SCFAs (PSCFAs) were calculated as the sum of isobutyric acid, isovaleric acid and valeric acid. All SCFAs analyses were performed in duplicate. Pure acetic, propionic, butyric, isobutyric, isovaleric and valeric acids were obtained from Sigma Co. (Poznan, Poland), and their mixture was used to create a standard plot and then to calculate the amount of single acids. This additional set of pure acids was included in each GC run of samples at five sample intervals to maintain calibration. In addition to SCFA analysis, cecal fermentation processes were analyzed based on the activities of selected bacterial enzymes (α- and β-glucosidase, α- and β-galactosidase, and β-glucuronidase), measured by the rate of release of ρ-nitrophenol or *o*-nitrophenol from the respective nitrophenyl glucosides, according to a previously described method [21]. The following substrates were used: ρ-nitrophenyl-α-d-glucopyranoside (for α-glucosidase), ρ-nitrophenyl-β-d-glucopyranoside (for β-glucosidase), ρ-nitrophenyl-α-d-galactopyranoside (for α-galactosidase), *o*-nitrophenyl-β-d-galactopyranoside (for β-galactosidase), and ρ-nitrophenyl-β-d-glucuronide (for β-glucuronidase). To measure the activities of enzymes secreted by bacterial cells into the cecal environment, we prepared a reaction mixture containing 0.3 mL of a substrate solution (5 mM) and 0.2 mL of a 1:10 (*v*/*v*) dilution of the cecal sample in 100 mM phosphate buffer (pH 7.0) after centrifugation at 7211× *g* for 15 min. Incubation was carried out at 37 °C, and ρ-nitrophenol was quantified at 400 nm (*o*-nitrophenol concentration—at 420 nm) after the addition of 2.5 mL of 0.25 M-cold sodium carbonate. Enzyme activity was expressed as μmol product formed per hour per g of digesta. To determine the total activity of selected cecal bacterial enzymes, including extracellular activity (see the procedure above) and intracellular activity, a cecal digesta sample diluted in phosphate buffer was mechanically disrupted by vortexing with glass beads (212–300 μm in diameter; four periods of 1 min with 1 min cooling intervals on ice) using the FastPrep^®^-24 homogenizer (MP Biomedicals, Santa Ana, CA, USA). The resulting mixture was centrifuged at 7211× *g* for 15 min at 4 °C. The supernatant was used for the enzyme assay described above. Intracellular enzyme activity was calculated by comparing total enzyme activity with the activities of bacterial enzymes secreted into the intestinal environment, and it was expressed as μmol product (ρ-nitrophenol (PNP) or *o*-nitrophenol (ONP)) formed per hour per g of digesta. To prepare the calculation formulas, the model curves for PNP and ONP (PNP or ONP standard solution in a 100 mM phosphate buffer pH 7.0, 40 mg/L) were used and appropriate equations obtained. Extracellular enzyme activity was determined as the rate of enzyme release, expressed as a percentage of total enzyme activity. All analyses were performed in duplicate.

Thiobarbituric acid-reactive substances (TBARS), which create lipid peroxidation, were determined in the heart, kidney and liver tissue after their storage at −70 °C. A procedure developed by Botsoglou et al. [22] was used in the assay, and the TBARS contents were determined spectrophotometrically at 532 nm and expressed in µg malondialdehyde per g of tissue. After storage of the liver, the reduced glutathione (GSH) and oxidized glutathione (GSSG) concentrations were determined by using an enzymatic recycling method described by Rahman et al. [23]. Liver lipids were extracted according to Folch et al. [24]. Following extraction, TC and TG concentrations were determined enzymatically using commercial kits (Cholesterol DST, Triglycerides DST, Alpha Diagnostics, Ltd., San Antonio, TX, USA). 

In the blood serum, the antioxidant capacity of water-soluble and lipid-soluble substances (ACW and ACL, respectively) was determined by a photochemiluminescence detection method using a Photochem device and the respective kits (ACW-Kit and ACL-Kit, Analytik Jena AG, Jena, Germany). In the photochemiluminescence assay, the generation of free radicals was partially eliminated through reactions with antioxidants present in the plasma samples, and the remaining radicals were quantified by luminescence generation. Ascorbate and Trolox calibration curves were used to evaluate ACW and ACL, respectively. Triglycerides (TG); total cholesterol (TC); fractions of HDL cholesterol (HDL) and LDL cholesterol (LDL); creatinine, urea, glucose, and fructosamine (FRC) concentrations; and activity of aspartate and alanine aminotransferases (AST and ALT, respectively) were estimated using a biochemical analyzer (Pentra C200, Horiba, Tokyo, Japan). An enzyme immunoassay (Cusabio Biotech Co., Ltd., Wuhan, Hubei, China) was used to determine the rat serum levels of adiponectin (rat adiponectin ELISA kit). To measure the insulin concentration, a validated rat insulin ELISA kit was used (Demeditec Diagnostics GmbH, Kiel, Germany). The serum concentrations of interleukin 6 (IL-6) and tumor necrosis factor-α (TNF-α) were determined using Thermo Scientific assays (Rockford, IL, USA). The HOMA-IR index was calculated according to the following formula: HOMA-IR = [fasting glycemia (mmol/L) × fasting insulinemia (mU/L)/22.5]. The atherogenic indices of plasma were calculated using the following formulas: Atherogenic Index I = lg (TG/HDL) and Atherogenic Index II = (TC − HDL)/HDL); values for TG, TC and HDL in mmol/L. 

### 2.4. Quantification of Ellagic Acid and ET Metabolites

The ellagic acid concentration was determined in the cecal digesta after their hydrolysis with trifluoroacetic acid. The digesta (0.2 mg) was mixed with 70% glycerol (0.5 mL) and 75 µL of trifluoroacetic acid and incubated at 95 °C for 18 h. Afterwards, the sample was cooled and extracted 3 times using 1.5 mL of methanol in an ultrasonic bath. After each extraction, the sample was centrifuged (3 min, 10,000× *g*), and the supernatant was collected in a volumetric flask and filled up with methanol. Ellagic acid was then determined using HPLC (Knauer Smartline system with photodiode array detector, Berlin, Germany) coupled with a Gemini C18 column (110 Å, 250 × 4.60 mm; 5 μm, Phenomenex, Torrance, CA, USA). Phase A was 0.05% phosphoric acid in water, Phase B was 0.05% phosphoric acid in 80% acetonitrile, the flow rate was 1.25 mL/min, the sample volume was 20 μL, and the temperature was 35 °C. The gradient was as follows: 10–25% B for 0–10 min, 25–40% B for 10–20 min, 40–80% B for 20–25 min, 80% B for 25–30 min, 80–10% B for 30–32 min, and 10% B for 32–40 min. The identification and quantification were performed at 360 nm with ellagic acid as a standard. 

The concentrations of ET metabolites were determined in the cecal digesta and urine. A frozen sample of the digesta (0.5–1 g) was mixed with acetone (2 mL), sonicated for 10 min, and centrifuged (5 min, 10,000× *g*); then, the supernatant was collected in a test tube. The procedure was repeated twice with 2 mL and 1 mL of 70% acetone. After collection of the supernatant, the extract was concentrated using a vacuum concentrator (ScanSpeed 40, LaboGene, Lillerød, Denmark) and then dissolved in methanol (1 mL). ET metabolites were then determined using HPLC (Knauer Smartline system with photodiode array detector, Berlin, Germany) coupled with a Gemini C18 column (110 Å, 250 × 4.60 mm; 5 μm, Phenomenex, Torrance, CA, USA). Separation conditions were the same as those used in the determination of ETs in dietary extracts. ET metabolites were identified by comparison of UV spectra with available literature data [25] and additionally confirmed by the MS method described below. A urine sample (0.5 mL) was mixed with acetone (1 mL), sonicated for 10 min, and centrifuged (5 min, 10,000× *g*); then, the supernatant was collected in a test tube. The procedure was repeated, and both supernatants were collected in a test tube and concentrated using a vacuum concentrator (ScanSpeed 40, LaboGene, Lillerød, Denmark). Next, the concentrated sample was dissolved in methanol (200 µL) and analyzed by HPLC-ESI-MS using a Dionex UltiMate 3000 UHPLC and a Thermo Scientific Q Exactive series quadrupole ion trap mass spectrometer. ET metabolites were separated using a Kinetex C18 column (110 Å, 150 × 2.1 mm; 2.6 μm, Phenomenex, Torrance, CA, USA) and a binary gradient of 0.1% formic acid in water (Phase A) and 0.1% formic acid in acetonitrile (Phase B) at a flow rate of 0.5 mL/min, as follows: stabilization for 1.44 min with 5% B, 5–15% B for 1.44 to 2.98 min, 15–40% B for 2.98–10.1 min, 40–73% B for 10.1–11.5 min, 73% B for 11.55–12.7 min, 73–5% B for 12.7–13.28 min, and 5% B for 13.28–18 min. The MS analysis was performed in negative ion mode under the following conditions: capillary voltage at +4 kV, sheath gas pressure at 75 arbitrary units, auxiliary gas at 17 arbitrary units, and scan range of 120–1200 *m*/*z*. Urolithin-A isolated from human urine by semipreparative HPLC was used as a standard for the quantification of ET metabolites. The detailed procedure of urolithin-A isolation is described elsewhere [26].

### 2.5. Statistical Analysis

The results are expressed as the means and pooled error of means (SEMs), except for the chemical composition of the strawberry extracts, which is expressed as the means ± standard deviations (SDs). A 2-factor analysis of variance (ANOVA) was used to determine the effect of the extract additions (E; none, monomeric ET-rich extract or dimeric ET-rich extract) and the diet type (D; corn starch and fructose as the main dietary carbohydrate) and the interaction between these two factors (E × D). If the analysis revealed a significant interaction (*p* ≤ 0.05), the differences among the respective treatment groups were then determined with Duncan’s post hoc test at *p* ≤ 0.05. The data were checked for normality prior to the statistical analyses. The statistical analysis was performed using STATISTICA software, version 10.0 (StatSoft Corp., Krakow, Poland).

## 3. Results

After the six-week feeding period, the dietary intake and growth rate of the animals did not differ among the groups (Table 2). In relation to the small intestine, the relative intestinal mass with digesta, pH value of the intestinal contents, and activities of mucosal disaccharidases maltase and lactase were significantly higher in rats fed fructose diets than in those on starch diets. An extract addition by diet type interaction (E × D) showed that mucosal sucrase activity was significantly increased only in rats fed a diet rich in fructose without the addition of a strawberry extract, and the values in groups F + ME and F + DE were comparable (*p* > 0.05) to those in groups C, C + ME and C + DE. Irrespective of diet type, dietary addition of the ME extract, but not the DE extract, significantly decreased the pH value of the small intestinal digesta. As shown in Table S2, the fructose treatment increased the accumulation rate of digesta (bulk effect) in the cecum and colon (*p* = 0.046 and *p* = 0.004 vs. starch, respectively).

The differences in the types of added strawberry extracts and dietary environment caused changes in the extra- and intracellular activities of certain bacterial enzymes in the cecal digesta of rats (Table 3 and Table 4). Irrespective of extract type, the extra- and intracellular activities of enzymes α- and β-glucosidase as well as α- and β-galactosidase were significantly reduced upon ingestion of dietary fructose. As a result, the calculated release rate of these enzymes from bacterial cells into the cecal environment was significantly enhanced in groups receiving diets high in fructose. In that context, the extract by diet type interactions were significant in terms of extracellular α-galactosidase (*p* = 0.016) as well as extra- and intracellular cecal activities of β-galactosidase (*p* = 0.002 and *p* = 0.001, respectively). The nature of these interactions involved a decrease in enzymatic activity in the cecum of rats fed diets containing the dimeric ET-rich extract (DE), and this decrease was especially noticeable among groups on starch-containing diets. Moreover, the two-way ANOVA revealed that the DE extract, especially due to effects on extracellular activity, significantly diminished the total activity of bacterial α-glucosidase, α-galactosidase, β-galactosidase, and β-glucuronidase in the cecum. The dietary monomeric ET-rich extract (ME) caused a significant increase in the total (including both extracellular and intracellular) activity of bacterial β-glucosidase (*p* < 0.05 vs. without extract and DE treatments, except intracellular activity *p* < 0.05 vs. without extract). As a result of the aforementioned changes in enzymatic activities, both strawberry extracts decreased the calculated release rate of α-galactosidase; the ME extract decreased the β-galactosidase release rate in the starch and fructose treatments (see significant E × D interaction), while the DE extract significantly lowered the release rate of bacterial β-glucosidase and β-glucuronidase (*p* < 0.05 vs. without extract and ME treatments and *p* < 0.05 vs. without extract treatment, respectively).

Regardless of the type of dietary carbohydrate, the addition of strawberry extracts influenced the SCFA concentration in the cecal digesta (Table 5). Both preparations caused an increase in the total cecal SCFA concentration, primarily due to their effect on the higher acetic acid level (*p* < 0.01 vs. without extract). The ME extract added to a diet significantly increased the cecal concentration of butyric acid (*p* < 0.05 vs. without extract and DE treatments). Compared with the starch carbohydrate type, dietary fructose significantly reduced the total SCFA concentration in the cecum (*p* = 0.002), due to the effect elicited by this dietary sugar in decreasing the levels of acetic, propionic and butyric acids. The profile analysis of three major short-chain fatty acids, i.e., acetic acid, propionic acid, and butyric acid, revealed that the dietary fructose treatment was characterized by a significantly greater acetic acid:total SCFA ratio (*p* = 0.020) and the opposite effect was noted when the butyric acid:total SCFA ratio was considered (*p* < 0.001). 

In both control groups, C and F, ellagitannin metabolites were absent; thus, Table 6 presents results representing four experimental groups treated with strawberry extracts. An extract by diet-type interaction showed that irrespective of the dietary fructose environment, both groups of rats consuming diets with the dimeric ET-rich extract were characterized by significantly lower nasutin-A concentrations in the cecal digesta in comparison to groups receiving the ME extract. When the C + ME and F + ME groups were considered, a significant decrease in the cecal nasutin-A concentration followed the fructose dietary treatment. The amounts of ellagic acid determined after hydrolysis in the cecal digesta were comparable in all experimental groups. The extract by diet-type interactions were significant for the nasutin-A glucuronide and nasutin-A concentrations in urine (*p* < 0.05). In the case of the former metabolite, the nature of this interaction was the same as that in the case of cecal nasutin-A levels. For urinary nasutin-A, both groups receiving monomeric ET-rich extracts had similar levels of that metabolite, while the F + DE group excelled the rats fed the starch-diet with DE extract. Irrespective of the diet type, the ME treatment was characterized by a significantly higher concentration of isonasutin-A in the urine, compared to the DE treatment (*p* < 0.001).

**Figure 1:**
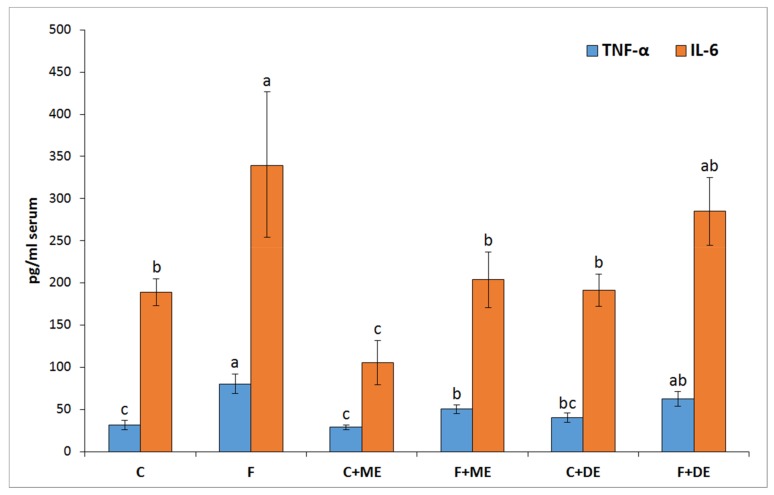
Pro-inflammatory cytokines in the blood serum of rats fed experimental diets. C, control fed a diet with 65.8% corn starch; F, fed a diet with 65.0% fructose (F) added at the expense of corn starch; C + ME, fed a corn starch-diet with a monomeric ET-rich extract; F + ME, fed a fructose-diet with a monomeric ET-rich extract; C + DE, fed a corn starch-diet with a dimeric ET-rich extract; F + DE, fed a fructose-diet with a dimeric ET-rich extract. ^a,b,c^ Mean values within a column with different superscript letters were shown to be significantly different (*p* < 0.05). Tumor necrosis factor-α, (TNF-α); Interleukin 6, (IL-6).

Irrespective of the experimental addition of the strawberry extracts, the applied dietary treatment with a high dosage of fructose caused several changes in biochemical blood serum parameters that should be considered not beneficial (Table 7), namely, an increase in ALT activity as well as in the concentration of glucose, fructosamine, insulin, and IL-6. The calculated HOMA-IR index was also significantly enhanced by dietary fructose (*p* < 0.001 vs. starch treatment). An extract by diet-type interaction indicated a significant increase in serum AST activity in the F group (*p* < 0.05 vs. all other groups) but not in groups F + ME and F + DE compared to their starch counterparts. Additionally, the dietary treatment with extract ME but not DE significantly reduced the serum concentrations of TNF-α and IL-6 in comparison to treatment without extract, irrespective of diet type (Figure 1). In relation to blood serum lipid indicators, some extract by diet-type interactions were found (Table 8). In the case of the serum total cholesterol concentration, the control fructose dietary treatment without addition of a strawberry extract caused a considerable rise in that parameter (*p* < 0.05 vs. all remaining groups). The serum levels of TC in groups F + ME and F + DE were also significantly higher than those of their starch counterparts, but the observed increase in the F + ME and F + DE groups was significantly lower than that in the F group (F > F + ME and F + DE at *p* < 0.05). The LDL cholesterol concentration in the serum was also associated with a significant extract by diet type interaction. Again, the F group was characterized by the highest LDL-C concentration (*p* < 0.05 vs. other groups), but, in groups C + ME and F + ME, the same serum concentrations of LDL-C were observed. This situation was not observed when comparing groups fed diets with dimeric ET-rich extract (F + DE > C + DE; *p* < 0.05). In the case of HDL-C profile values, an extract by diet-type interaction showed that the F group had the lowest value (*p* < 0.05 vs. all other groups), and the values calculated for groups C + ME, F + ME, C + DE but not for F + DE were statistically insignificant compared to values calculated for the control C rats. The dietary fructose treatment was associated with a significant lower concentration of adiponectin and higher concentration of triglycerides than the starch treatment. The two-way ANOVA showed that the calculated atherogenic index I of the serum, expressed as lg(TG/HDL), was significantly increased by dietary fructose (irrespective of extract addition) and was significantly decreased by the ME treatment (irrespective of diet type; *p* < 0.05 vs. treatments without extract and DE). In relation to the serum atherogenic index II, calculated with the aid of the (TC-HDL)/HDL equation, an extract by diet-type interaction showed that the highest value of the serum atherogenic index II was noted in the F group (*p* < 0.05 vs. other groups). The rats fed C + ME and F + ME diets had comparable values for that index, even in comparison to the control rats fed starch diet (*p* > 0.05). When the C + DE and F + DE groups were compared, the latter had a significantly higher value in relation to the former.

Irrespective of diet type, the administration of extract ME caused a significant increase in the concentration of serum ACL in comparison to other treatments, i.e., without any extract addition and with extract DE (Table 9). The concentration of serum ACW was modified by both the type of diet and extract addition, and there was an interaction between E and D main factors (*p* = 0.002). The highest ACW value was noted in the C + ME group (*p* < 0.05 vs. all other groups), but the F + ME rats were characterized by an ACW level comparable (*p* > 0.05) to those of groups C and F, in which the lowest ACW values were noted. Interestingly, the rats fed the fructose diet with extract DE (group F + DE) had an ACW level significantly higher than not only the levels in C and F animals but also the levels in group F + ME. In the liver, the concentrations of TG and TC were affected by an interaction extract by diet type (*p* = 0.001 and *p* < 0.001, respectively). For the TG level in the liver, the rats fed the fructose diet with extract ME had a significantly lower TG concentration than the control fructose group; such effects were not noted when groups F and F + DE were compared. In the case of the liver TC concentration, both fructose groups with ME or DE extract addition (F + ME and F + DE groups, respectively) were characterized by a significantly decreased total cholesterol concentration in the liver in comparison to the concentrations in F rats. All groups receiving the starch diet with or without extract addition did not differ from each other in respect to liver concentrations of TG and TC. An extract by diet-type interaction was also noted in liver GSSG concentrations as well as when the GSH:GSSG ratio was considered. The highest GSSG level in the liver was measured in the F group (*p* < 0.005 vs. all other groups). There was a significant difference between the C + DE and F + DE rats in relation to the GSSG liver concentration (F + DE > C + DE at *p* < 0.05), and no significant changes in that parameter were found when groups C + ME and F + ME were compared. The liver GSH:GSSG ratio was significantly decreased in the F group in comparison to the C group; when groups C + ME and F + ME as well as groups C + DE and F + DE were compared, the observed differences between these two pairs of counterparts did not reach statistical significance (*p* > 0.05).

## 4. Discussion

Diet modification by additional fructose did not affect the growth rate of rats and their food intake but led to some undesired effects in gastrointestinal functioning, as manifested, for instance, by increased activities of mucosal disaccharidases (sucrase, maltase and lactase) in the small intestine and decreased activities of bacterial enzymes (except for the potentially harmful activity of β-glucuronidase) in the cecal digesta. The former changes should be considered as contributors to the observed rise in blood glucose, FRC and HOMA-IR values, while the latter caused lower formation of fermentation products such as SCFAs, which are important energy sources for the large gut epithelium [27]. The extracellular activities of cecal bacterial enzymes, i.e., those released from the microorganisms into the cecal environment, directly affect the rate of bacterial digestion of nutrients and non-nutrients in lower parts of the gastrointestinal tract [28]. Extracellular activity of an enzyme is related to the types and counts of microorganism species colonizing the intestine, i.e., the ability of certain bacteria to produce that enzyme and to the rate of its secretion from cells into outside environment. In the present study, rats on the fructose dietary treatment were characterized by a lower total (including extra- and intracellular) activity of α- and β-glucosidase as well as α- and β-galactosidase in comparison to rats fed starch diets. This effect may indirectly point to a considerably lower density of cecal bacteria upon a large dietary load of fructose. It has been reported that high-fructose diets should be considered unfriendly treatments to the lower parts of the gastrointestinal tract [26], and one of the physiological mechanisms that can help to obtain additional energy and optimize the bacterial digestion processes would be an enhanced release rate of enzymes from bacterial cells to the digesta. Indeed, the release rate (i.e., extracellular activity expressed as a percentage of total activity) of all cecal enzymes measured in the present study was significantly increased in fructose-fed rats. 

Polyphenols, including ET, are well-known dietary ingredients that modulate the activity of intestinal microbiota and may affect the formation of anaerobic bacterial fermentation products, such as ammonia and SCFA [15,20,26]. In the present study, the dietary application of strawberry extract DE (containing mainly dimeric ETs) exerted a lowering effect on bacterial enzymatic activity in the rat cecum, thus indicating stronger antimicrobial properties than extract ME (containing mainly ellagitannin monomers). The treatment with extract DE was accompanied by a significantly lower total and extracellular activity of most of the analyzed enzymes, which supported the hypothesis that dimeric ETs are more active as antibacterial agents than ETs with a lower degree of polymerization. Of course, in the case of the DE extract, the additional effects of proanthocyanidins on bacterial enzymatic activity cannot be excluded. In our opinion, the main action should be ascribed to different ellagitannins because the proanthocyanidin fraction was also present in the ME extract, however, to a lesser extent than in the DE extract. Our previous study on rats [29] showed that an additional dose of dietary proanthocyanidins (0.01% of a diet) in a diet with 0.03% strawberry ET did not affect the levels of bacterial glycolytic activity in the cecum of rats (the same enzymes were investigated as in the present study). In a recent experiment on rats, Kosmala et al. [15] reported changes caused by strawberry and raspberry polyphenols on cecal bacterial enzymatic activity depending on the degree of polymerization of ET. Those authors observed considerably lower cecal enzymatic activity upon dietary addition of a raspberry preparation containing sanguiin-H6 (dimer) and lambertianin-C (trimer) in its polyphenolic fraction, in comparison to the effects caused by a strawberry preparation with monomers and agrimoniin (dimer) as the dominant ET. The activities of large gut microbiota have direct impacts on ET metabolism, then on the appearance of ET metabolites (urolithins, nasutins, and their conjugates) in intestinal digesta, and finally on blood, tissues, and urine; thus, some systemic modulatory effects of ET derivatives may be observed [14,16,30,31]. On the other hand, there are also some reports about greater or lower susceptibility of various ETs with different degrees of polymerization to intestinal breakdown and the appearance of systemic metabolites [14,15]. The aforementioned authors found that the lower the degree of polymerization of ETs, the more ETs are prone to bacterial breakdown in the intestine, and the higher the amount of metabolites present in the cecal digesta of rats. The results from the present study were in line with the abovementioned findings, as the ME treatment was characterized by an almost four-fold higher concentration of nasutin-A in the cecal digesta than treatment DE. Nevertheless, although 1.4–1.8 mg ETs were present in a gram of the experimental diets, the concentration of nasutin-A in the cecal contents was on the level of 10–40 µg per gram of digesta, while large quantities of ellagic acid were released from ET molecules after hydrolysis of the digesta (1.4–1.5 mg/g). These results indicated a very slow and limited metabolism of dietary ET, but, surprisingly, the observed concentration of ET metabolites in the urine was relatively high, especially in the ME treatment (ca. 1000 µg per mL of urine), which supported the hypothesis that in comparison to dimeric ETs, monomeric ones are more prone to intestinal breakdown and provide greater amounts of metabolites reaching the internal tissues of the host. Some authors have reported that urolithins are considered as the only bacterial bioavailable metabolites of ETs found in rats and humans intestinal digesta, plasma and urine [5,16,32,33]. In fact, our team findings taken not only from the present experiment but also from other authors’ data suggested that both urolithins and nasutins might be found in the body after ET ingestion [14,15,25,26]. The reasons that researchers sometimes detected only urolithins or nasutins are: (i) differences in the chemical structures of the ETs consumed (ETs from the strawberry, raspberry, pomegranate, etc.); (ii) different samples analyzed (intestinal digesta, feces, blood, urine, and tissues); and (iii) different intestinal microbiota compositions and readiness to metabolize polyphenols (which is also affected by other non-polyphenolic dietary ingredients). In the present study, the main urinary metabolites found were glucuronidated nasutin-A and, in lesser concentrations, unconjugated forms of nasutin-A and isonasutin-A. The glucuronidation process of metabolites increases their water solubility and facilitates their excretion in urine [14,34]. 

There are many reports providing information about positive changes in body health status following the consumption of fresh fruit and preparations or extracts obtained from strawberries that indicate fiber and polyphenols as the main biologically active fractions [12,15,30,35,36]. The present paper lists some physiological changes upon ingestion of dietary strawberry extracts, but, to the best of our knowledge, it presents new results regarding the biological action of ETs with different degrees of polymerization. Food modification by additional dietary fructose, as expected, led to some adverse systemic effects in the current experiment. In rats, a high-fructose treatment disturbed the lipid and glucose metabolism as well as antioxidant status of the body within a few weeks. It has been reported that liver and kidney hypertrophy is commonly associated with a great load of ingested fructose [37,38], and such effects were observed in our study. Fructose is avidly metabolized in the liver in a highly lipogenic manner causing increased fat accumulation, while, in kidneys, high fructose consumption leads to an increase in the weight of the cortex and hyperuricemia [36,39,40]. In our previous study of rats fed for four weeks with diets high in fructose [36], some metabolic perturbations associated with metabolic syndrome or diabetes were partially reversed by the dietary addition of strawberry pomaces (7.7% of a diet), and more beneficial effects were observed in animals fed a polyphenol-rich pomace than in those fed a preparation deprived of most of these compounds via water/ethanol extraction. In the current experiment, the addition of strawberry extracts to a diet considerably reduced the relative renal mass in fructose-fed rats, though no changes in serum creatinine and urea concentrations were noted. It should be stressed that during the entire study, the technical staff reported an evident reduction in the volume of urine excreted in the rats from the F + ME and F + DE groups in comparison to the F rats. Comparing both extracts, the ME one induced favorable changes in the redox state of the kidneys as manifested by a significantly lower level of kidney TBARS in relation to the control diets. Such changes could be ascribed to the levels of urinary metabolites of ET and EA, which were much higher in rats fed diets containing extract ME than in those fed diets with DE. Mazzone et al. [41], after computations performed in the gas phase and in both water and methanol media in relation to the antioxidant ability of ellagic acid and some of its derivatives, reported the latter as the most promising candidates as antioxidants. 

In the present study, despite the unreduced relative liver mass, the dietary addition of both strawberry polyphenolic extracts partially prevented the accumulation of TC and TG in liver tissue. Moreover, both strawberry extract treatments (especially in the dietary fructose environment) were accompanied by health-promoting changes in serum TC, LDL, and TG concentrations; serum AST activity; serum ACW and ACL values; and liver GSSG concentration, with the latter resulting in a desirable increase in the GSH:GSSG ratio. At this point, it should be underlined that the stronger antioxidant and antihyperlipidemic action of strawberry polyphenols was noticeable for the extract abundant in monomeric ETs, in comparison to the DE extract. Again, these effects at least could be ascribed to the greater amounts of ET metabolites that can reach internal tissues, as was shown in this study in the case of the bladder. Recently, Saha et al. [12] provided new data about the potent antioxidant action of urolithin A (UA) derived via microbiota-mediated conversion of EA. These authors demonstrated that UA potently inhibited heme peroxidases i.e., myeloperoxidase and lactoperoxidase in comparison to the parent compound EA, and a clear conclusion was formed that intestinal microbiota-derived metabolites were critical in controlling inflammatory pathways. Indeed, in the current study, the ME treatment, but not the DE one, efficiently reduced the serum concentration of TNF-α and IL-6. This result supports the accepted hypothesis that dietary monomeric- and dimeric-rich extracts would beneficially but to some extent differentially alleviate disturbances associated with high fructose administration. The role of stimulatory cytokines in excessive B-cell activation has been reported for many diseases, and several cytokines have been implicated as important mediators, including IL-10, IL-6 and TNF-α [42]. Moreover, some authors identified IL-6 as a target protein for the TNF-α signaling pathway that regulates the cell inflammatory response [43]. 

## 5. Conclusions

Concerning the present study’s hypothesis, the six-week consumption of the fructose diet with the addition of a monomeric ET-rich strawberry extract exerted more favorable modifications leading to a decreased lipid peroxidation level in some tissues, a healthier serum and liver lipid profile, and a greater anti-inflammatory effect in blood serum, in comparison to the dimeric ET-rich extract. Partly, these effects could be ascribed to substantially greater amounts of metabolites derived from monomeric ETs that appeared in the cecal digesta and internal body fluids (in this study, found in urine), proving that monomeric ETs are more prone to bacterial breakdown than dimeric ET. The results clearly indicate that a relatively small difference in the degree of ET polymerization may cause different physiological responses, which should be considered when assessing the biological properties of fruit ellagitannins. Nevertheless, it is well known that metabolism of the ETs is different between animal models and human, therefore in the future studies it is needed to confirm regulatory effect of the ET-rich extracts with different degree of polymerization on physiological function of the gastrointestinal tract as well as systemic parameters in human trials.

## Figures and Tables

**Table 1:** Chemical composition of the strawberry ET extracts.

	Monomeric ET-Rich Extract (g/100 g)	Dimeric ET-Rich Extract (g/100 g)
Dry matter	94.31 ± 0.25	91.3 ± 0.05
Ash	0.34 ± 0.02	0.03 ± 0.04
Fat	-	-
Protein	5.62 ± 0.05	1.83 ± 0.03
Other components ^1^	0.35 ± 0.01	7.17 ± 0.01
Total polyphenols (HPLC-DAD)	88.0 ± 0.10	82.3 ± 0.1
Ellagic acid	0.1 ± 0.0	0.2 ± 0.0
Ellagitannins (ET)	80.0 ± 0.1	57.3 ± 0.1
Monomers	77.1 ± 0.1	23.3 ± 0.1
Dimers	2.9 ± 0.0	34.0 ± 0.1
Proanthocyanidins	8.1 ± 0.3	23.9 ± 0.2
Flavonols	-	0.9 ± 0.0
Anthocyanins	-	-

The results are expressed as the mean ± SD, *n* = 2; ^1^ Low-molecular carbohydrates and structural components of plant cell walls, including dietary fiber.

**Table 2:** Growth parameters and small intestinal indices of rats fed experimental diets *.

	Start BW	Gain	Food Intake	Small Intestine				
	g	g	g	Mass ^1^	pH	Sucrase ^2^	Maltase ^2^	Lactase ^2^
Group (*n* = 8)								
C	176	170	647	1.96	7.29	6.69 ^b^	42.4	1.78
F	176	170	623	2.12	7.62	10.3 ^a^	51.6	2.87
C + ME	175	164	648	1.92	7.01	6.55 ^b^	40.7	1.94
F + ME	175	162	637	2.09	7.38	7.23 ^b^	43.0	2.62
C + DE	175	159	656	1.91	7.29	7.43 ^b^	41.1	2.24
F + DE	176	155	652	2.17	7.45	7.60 ^b^	46.9	2.74
*SEM*	*1.251*	*2.619*	*9.045*	*0.027*	*0.047*	*0.260*	*1.268*	*0.094*
Extract (E)								
- (without)	176	170	635	2.04	7.46 ^a^	8.49 ^a^	47.0	2.32
ME	175	163	643	2.01	7.19 ^b^	6.89 ^b^	41.8	2.23
DE	175	157	654	2.04	7.37 ^a,b^	7.51 ^b^	44.0	2.49
*p value*	*NS*	*NS*	*NS*	*NS*	*0.033*	*0.007*	*NS*	*NS*
Diet (D)								
Corn starch	175	164	650	1.93 ^b^	7.20 ^b^	6.89 ^b^	41.4 ^b^	1.95 ^b^
Fructose	175	162	637	2.13 ^a^	7.48 ^a^	8.37 ^a^	47.2 ^a^	2.74 ^a^
*p value*	*NS*	*NS*	*NS*	<*0.001*	*0.001*	*0.001*	*0.021*	<*0.001*
Interaction E × D								
*p value*	*NS*	*NS*	*NS*	*NS*	*NS*	*0.002*	*NS*	*NS*

* C, control fed a diet with 65.8% corn starch; F, fed a diet with 65.0% fructose (F) added at the expense of corn starch; C + ME, fed a corn starch-diet with a monomeric ET-rich extract; F + ME, fed a fructose-diet with a monomeric ET-rich extract; C + DE, fed a corn starch-diet with a dimeric ET-rich extract; F + DE, fed a fructose-diet with a dimeric ET-rich extract. ^a,b^ Mean values within a column with different superscript letters were shown to be significantly different (*p* < 0.05); differences among the groups (C, F, C + ME, F + ME, C + DE, and F + DE) are indicated with superscripts only in the case of a statistically significant interaction E × D (*p* < 0.05). ^1^ mass with contents, g/100 g BW; ^2^ µmol/min/g protein; BW, body weight.

**Table 3:** Cecal bacterial enzyme activity and their release rate into the intestinal environment in rats *.

	α-Glucosidase				α-Galactosidase				β-Galactosidase			
	Extracellular	Intracellular	Total	Release Rate	Extracellular	Intracellular	Total	Release Rate	Extracellular	Intracellular	Total	Release Rate
	µmol/h/g Digesta			%	µmol/h/g Digesta			%	µmol/h/g Digesta			%
Group (*n* = 8)												
C	10.6	2.40	13.0	81.8	9.79 ^a^	2.68	12.5	78.6	28.5 ^a^	9.81 ^a^	38.3 ^a^	75.3 ^b^
F	6.91	0.65	7.56	91.6	6.18 ^b^	1.03	7.21	85.4	17.5 ^b,c^	1.46 ^c^	18.9 ^c^	92.1 ^a^
C + ME	9.48	2.87	12.4	76.7	8.95 ^a^	3.81	12.8	71.8	26.1 ^a^	12.1 ^a^	38.2 ^a^	68.5 ^c^
F + ME	6.49	0.75	7.14	90.4	5.96 ^b^	2.52	8.48	71.8	20.5 ^b^	6.09 ^b^	26.6 ^b^	77.5 ^b^
C + DE	7.55	2.44	9.99	75.7	6.08 ^b^	2.63	8.71	69.8	14.2 ^c^	2.24 ^c^	16.4 ^c^	85.9 ^a^
F + DE	5.95	0.45	6.40	92.8	5.64 ^b^	1.47	7.11	81.4	15.9 ^b,c^	1.48 ^c^	17.4 ^c^	91.4 ^a^
*SEM*	*0.309*	*0.167*	*0.440*	*1.243*	*0.320*	*0.220*	*0.476*	*1.367*	*0.998*	*0.704*	*1.569*	*1.539*
Extract (E)												
- (without)	8.78 ^a^	1.52	10.3 ^a^	86.7	7.98 ^a^	1.86 ^b^	9.84 ^a^	82.0 ^a^	23.0 ^a^	5.63 ^b^	28.6 ^a^	83.7 ^b^
ME	7.98 ^a^	1.81	9.80 ^a^	83.5	7.45 ^a^	3.16 ^a^	10.6 ^a^	71.8 ^b^	23.3 ^a^	9.07 ^a^	32.4 ^a^	73.0 ^c^
DE	6.75 ^b^	1.45	8.20 ^b^	84.2	5.96 ^b^	2.05 ^b^	7.91 ^b^	75.6 ^b^	15.0 ^b^	1.86 ^c^	16.9 ^b^	88.7 ^a^
*p value*	*0.001*	*NS*	*0.003*	*NS*	*0.001*	*0.014*	*0.010*	*0.003*	<*0.001*	<*0.001*	<*0.001*	<*0.001*
Diet (D)												
Corn starch	9.22 ^a^	2.57 ^a^	11.8 ^a^	78.0 ^b^	8.27 ^a^	3.04 ^a^	11.3 ^a^	73.4 ^b^	22.9 ^a^	8.04 ^a^	31.0 ^a^	76.6 ^b^
Fructose	6.45 ^b^	0.62 ^b^	7.07 ^b^	91.6 ^a^	5.92 ^b^	1.68 ^b^	7.60 ^b^	79.5 ^a^	18.0 ^b^	3.01 ^b^	21.0 ^b^	87.0 ^a^
*p value*	<*0.001*	<*0.001*	<*0.001*	<*0.001*	<*0.001*	*0.014*	*0.010*	*0.010*	*0.001*	<*0.001*	<*0.001*	<*0.001*
Interaction E × D												
*p value*	*NS*	*NS*	*NS*	*NS*	*0.016*	*NS*	*NS*	*NS*	*0.002*	*0.001*	<*0.001*	*0.046*

* C, control fed a diet with 65.8% corn starch; F, fed a diet with 65.0% fructose (F) added at the expense of corn starch; C + ME, fed a corn starch diet with a monomeric ET-rich extract; F + ME, fed a fructose diet with a monomeric ET-rich extract; C + DE, fed a corn starch diet with a dimeric ET-rich extract; F + DE, fed a fructose diet with a dimeric ET-rich extract. ^a,b,c^ Mean values within a column with different superscript letters were shown to be significantly different (*p* < 0.05); differences among the groups (C, F, C + ME, F + ME, C + DE, and F + DE) are indicated with superscripts only in the case of a statistically significant interaction E × D (*p* < 0.05). Release rate, extracellular expressed as percent of total activity.

**Table 4:** Activity of cecal bacterial enzymes and their release rate into the intestinal environment in rats *.

	β-Glucosidase				β-Glucuronidase			
	Extracellular	Intracellular	Total	Release Rate	Extracellular	Intracellular	Total	Release Rate
	µmol/h/g Digesta			%	µmol/h/g Digesta			%
Group (*n* = 8)								
C	2.10	0.70	2.80	74.6	11.8	5.63	17.4	68.6
F	0.73	0.15	0.88	83.2	17.4	4.34	21.7	80.1
C + ME	3.33	1.73	4.82	72.0	13.8	6.53	20.3	66.2
F + ME	2.55	0.57	3.13	82.9	15.4	4.22	19.6	78.5
C + DE	1.74	1.27	3.01	57.9	7.49	4.40	11.9	63.4
F + DE	1.26	0.62	1.88	66.1	8.84	3.60	12.4	72.2
*SEM*	*0.164*	*0.102*	*0.241*	*2.046*	*0.660*	*0.273*	*0.765*	*1.355*
Extract (E)								
- (without)	1.41 ^b^	0.42 ^b^	1.83 ^b^	78.9 ^a^	14.6 ^a^	4.99	19.6 ^a^	74.3 ^a^
ME	2.94 ^a^	1.03 ^a^	3.97 ^a^	77.5 ^a^	14.6 ^a^	5.37	20.2 ^a^	72.3 ^a,b^
DE	1.50 ^b^	0.94 ^a^	2.45 ^b^	62.0 ^b^	8.16 ^b^	4.00	12.2 ^b^	67.8 ^b^
*p value*	<*0.001*	*0.009*	<*0.001*	<*0.001*	<*0.001*	*NS*	<*0.001*	*0.042*
Diet (D)								
Corn starch	2.39 ^a^	1.15 ^a^	3.54 ^a^	68.1 ^b^	11.3 ^b^	5.52 ^a^	16.6	66.1 ^b^
Fructose	1.52 ^b^	0.45 ^b^	1.96 ^b^	77.4 ^a^	13.9 ^a^	4.05 ^b^	17.9	76.9 ^a^
*p value*	<*0.001*	<*0.001*	<*0.001*	*0.008*	*0.003*	*0.005*	*NS*	<*0.001*
Interaction E × D								
*p value*	*NS*	*NS*	*NS*	*NS*	*NS*	*NS*	*NS*	*NS*

* C, control fed a diet with 65.8% corn starch; F, fed a diet with 65.0% fructose (F) added at the expense of corn starch; C + ME, fed a corn starch diet with a monomeric ET-rich extract; F + ME, fed a fructose diet with a monomeric ET-rich extract; C + DE, fed a corn starch diet with a dimeric ET-rich extract; F + DE, fed a fructose diet with a dimeric ET-rich extract. ^a,b^ Mean values within a column with different superscript letters were shown to be significantly different (*p* < 0.05); differences among the groups (C, F, C + ME, F + ME, C + DE, and F + DE) are indicated with superscripts only in the case of a statistically significant interaction E × D (*p* < 0.05). Release rate, extracellular expressed as percent of total activity.

**Table 5:** Short-chain fatty acid (SCFA) concentration and profile in the cecal digesta of rats fed experimental diets *.

	SCFA Concentration, μmol/g Digesta								SCFA Profile, Percent of Total SCFA		
	C2	C3	C4i	C4	C5i	C5	PSCFA	SCFA	C2	C3	C4
Group (*n* = 8)											
C	94.1	22.2	2.11	15.4	2.47	2.07	6.65	138	67.7	16.2	11.2
F	73.1	13.8	1.82	7.36	2.50	2.31	6.63	101	72.2	13.8	7.32
C + ME	101	23.0	2.14	16.3	2.16	2.51	6.81	147	68.6	15.7	11.0
F + ME	104	20.8	2.47	13.0	2.59	2.63	7.70	146	71.3	14.4	8.98
C + DE	104	21.7	3.03	14.7	3.11	2.74	8.88	149	69.7	14.5	9.81
F + DE	90.4	19.6	4.07	10.2	1.92	2.35	8.33	128	70.8	15.1	7.97
*SEM*	*2.639*	*0.757*	*0.327*	*0.617*	*0.162*	*0.091*	*0.413*	*3.722*	*0.575*	*0.360*	*0.310*
Extract (E)											
- (without)	83.6 ^b^	18.0 ^b^	1.96	11.4 ^b^	2.49	2.19	6.64	120 ^b^	70.0	15.0	9.26
ME	103 ^a^	21.9 ^a^	2.30	14.6 ^a^	2.38	2.57	7.25	146 ^a^	69.9	15.0	10.0
DE	97.2 ^a^	20.6 ^a,b^	3.55	12.4 ^b^	2.51	2.54	8.61	135 ^a^	70.3	14.8	8.89
*p value*	*0.004*	*NS*	*NS*	*0.013*	*NS*	*NS*	*NS*	*0.002*	*NS*	*NS*	*NS*
Diet (D)											
Corn starch	99.7 ^a^	22.3 ^a^	2.43	15.5 ^a^	2.58	2.44	7.45	145 ^a^	68.7 ^b^	15.5	10.7 ^a^
Fructose	89.2 ^b^	18.1 ^b^	2.78	10.2 ^b^	2.34	2.43	7.55	125 ^b^	71.4 ^a^	14.4	8.09 ^b^
*p value*	*0.024*	*0.002*	*NS*	<*0.001*	*NS*	*NS*	*NS*	*0.002*	*0.020*	*NS*	<*0.001*
Interaction E × D											
*p value*	*NS*	*NS*	*NS*	*NS*	*NS*	*NS*	*NS*	*NS*	*NS*	*NS*	*NS*

* C, control fed a diet with 65.8% corn starch; F, fed a diet with 65.0% fructose (F) added at the expense of corn starch; C + ME, fed a corn starch diet with a monomeric ET-rich extract; F + ME, fed a fructose diet with a monomeric ET-rich extract; C + DE, fed a corn starch diet with a dimeric ET-rich extract; F + DE, fed a fructose diet with a dimeric ET-rich extract. ^a,b^ Mean values within a column with different superscript letters were shown to be significantly different (*p* < 0.05); differences among the groups (C, F, C + ME, F + ME, C + DE, and F + DE) are indicated with superscripts only in the case of a statistically significant interaction E × D (*p* < 0.05). PSCFA, putrefactive SCFA (the sum of isobutyric, isovaleric and valeric acids); acids: C2, acetic; C3, propionic; C4i, isobutyric; C4, butyric; C5i, isovaleric; C5, valeric.

**Table 6:** Ellagitannin metabolite profile in the cecal digesta and in the urine of rats fed experimental diets *.

	Cecum		Urine		
	Nasutin A ^1^	Released EA	Nasutin A glucuronide ^2^	Nasutin A ^1^	Isonasutin A ^3^
	µg/g	mg/g	µg/mL	µg/mL	µg/mL
Group (*n* = 8)					
C + ME	43.1 ^a^	1.42	1305 ^a^	76.5 ^a^	83.2
F + ME	30.6 ^b^	1.52	824 ^b^	60.4 ^a,b^	82.6
C + DE	10.4 ^c^	1.51	360 ^c^	41.8 ^b^	12.5
F + DE	10.6 ^c^	1.47	369 ^c^	68.0 ^a^	19.5
*SEM*	*2.905*	*0.039*	*94.51*	*5.100*	*7.678*
Extract (E)					
ME	36.8 ^a^	1.47	1064 ^a^	68.5	82.9 ^a^
DE	10.5 ^b^	1.49	365 ^b^	54.9	16.0 ^b^
*p value*	<*0.001*	*NS*	<*0.001*	*NS*	<*0.001*
Diet (D)					
Starch	26.8	1.47	832	59.2	47.8
Fructose	20.6	1.50	597	64.2	51.0
*p value*	*NS*	*NS*	*NS*	*NS*	*NS*
Interaction E × D					
*p value*	*0.048*	*NS*	*0.047*	*0.036*	*NS*

* C + ME, fed a corn starch diet with a monomeric ET-rich extract; F + ME, fed a fructose diet with a monomeric ET-rich extract; C + DE, fed a corn starch diet with a dimeric ET-rich extract; F + DE, fed a fructose diet with a dimeric ET-rich extract. ^a,b,c^ Mean values within a column with different superscript letters were shown to be significantly different (*p* < 0.05); differences among the groups (C + ME, F + ME, C + DE, and F + DE) are indicated with superscripts only in the case of a statistically significant interaction E × D (*p* < 0.05). ^1^ HPLC retention time (min) 10.08; MS [M − H]^−^ 269; MS/MS fragments 113, 85.03; UV spectra (nm) 227, 245, 284, 323, 389. ^2^ HPLC retention time (min) 6.99; MS [M − H]^−^ 445; MS/MS fragments 269.01, 113, 85.03; UV spectra (nm) 222, 279, 315, 367, 379. ^3^ HPLC retention time (min) 10.21; MS [M − H]^−^ 269; MS/MS fragments 113, 85.03; UV spectra (nm) 227, 245, 284, 323, 389.

**Table 7:** Biochemical indicators in the blood serum of rats fed experimental diets *.

	AST	ALT	Creatinine	Urea	Glucose	FRC	Insulin	HOMA-IR
	U/L	U/L	µmol/L	mmol/L	mmol/L	µmol/L	pmol/L	
Group (*n* = 8)								
C	65.5 ^b^	22.8	18.5	4.78	9.60	160	28.8	1.78
F	84.0 ^a^	44.3	18.9	5.13	13.9	172	38.3	3.47
C + ME	70.0 ^b^	57.8	18.1	4.87	9.94	163	25.8	1.64
F + ME	67.7 ^b^	41.7	17.8	5.13	12.5	167	35.2	2.81
C + DE	67.9 ^b^	27.5	18.0	4.88	10.6	160	28.2	1.79
F + DE	71.0 ^b^	36.0	18.7	4.96	13.0	172	37.7	3.12
*SEM*	*2.013*	*1.685*	*0.519*	*0.090*	*0.308*	*1.671*	*1.694*	*0.153*
Extract (E)								
- (without)	74.8	33.5	18.7	4.95	11.8	166	33.6	2.62
ME	68.9	34.7	17.9	5.00	11.2	165	30.5	2.22
DE	69.4	31.8	18.3	4.92	11.8	166	30.3	2.46
*p value*	*NS*	*NS*	*NS*	*NS*	*NS*	*NS*	*NS*	*NS*
Diet (D)								
Corn starch	67.8	26.0 ^b^	18.2	4.84	10.0 ^b^	161 ^b^	27.6 ^b^	1.74 ^b^
Fructose	74.2	40.7 ^a^	18.5	5.07	13.1 ^a^	170 ^a^	37.1 ^a^	3.13 ^a^
*p value*	*NS*	<*0.001*	*NS*	*NS*	<*0.001*	*0.008*	*0.006*	<*0.001*
Interaction E × D								
*p value*	*0.047*	*NS*	*NS*	*NS*	*NS*	*NS*	*NS*	*NS*

* C, control fed a diet with 65.8% corn starch; F, fed a diet with 65.0% fructose (F) added at the expense of corn starch; C + ME, fed a corn starch diet with a monomeric ET-rich extract; F + ME, fed a fructose diet with a monomeric ET-rich extract; C + DE, fed a corn starch diet with a dimeric ET-rich extract; F + DE, fed a fructose diet with a dimeric ET-rich extract. ^a,b^ Mean values within a column with different superscript letters were shown to be significantly different (*p* < 0.05); differences among the groups (C, F, C + ME, F + ME, C + DE, and F + DE) are indicated with superscripts only in the case of a statistically significant interaction E × D (*p* < 0.05). ALT, alanine transaminase; AST, aspartate transaminase; ALP, alkaline phosphatase; FRC, fructosamine; HOMA-IR = (fasting insulin (mU/L) × fasting glucose (mmol/L)/22.5).

**Table 8:** Biochemical indicators in the blood serum lipid profile of rats fed experimental diets *.

	Adiponectin	TC	HDL	LDL	HDL Profile	TG	Atherogenic Index I	Atherogenic Index II
	ng/mL	mmol/L	mmol/L	mmol/L	% of TC	mmol/L	lg(TG/HDL)	(TC-HDL)/HDL
Group (*n* = 8)								
C	894	2.26 ^c^	0.629	0.444 ^c^	27.8 ^a,b^	2.15	0.530	2.61 ^c,d^
F	777	3.41 ^a^	0.675	0.651 ^a^	19.9 ^d^	3.41	0.695	4.06 ^a^
C + ME	880	2.35 ^c^	0.636	0.503 ^b,c^	27.1 ^a,b^	1.63	0.484	2.75 ^c,d^
F + ME	860	2.74 ^b^	0.673	0.504 ^b,c^	24.8 ^b,c^	2.89	0.714	3.09 ^b,c^
C + DE	873	2.28 ^c^	0.673	0.460 ^c^	29.7 ^a^	2.12	0.397	2.42 ^d^
F + DE	827	2.84 ^b^	0.679	0.518 ^b^	23.9 ^c^	3.46	0.635	3.25 ^b^
*SEM*	*16.88*	*0.079*	*0.014*	*0.020*	*0.619*	*0.125*	*0.020*	*0.102*
Extract (E)								
- (without)	836	2.84	0.652	0.548	23.9 ^b^	2.78 ^a^	0.612 ^a^	3.34 ^a^
ME	870	2.55	0.654	0.503	25.9 ^a,b^	2.26 ^b^	0.599 ^a^	2.92 ^b^
DE	850	2.56	0.676	0.489	26.8 ^a^	2.79 ^a^	0.516 ^b^	2.83 ^b^
*p value*	*NS*	*NS*	*NS*	*NS*	*0.029*	*0.012*	*0.005*	*0.010*
Diet (D)								
Corn starch	882 ^a^	2.30 ^b^	0.646	0.469 ^b^	28.2 ^a^	1.96 ^b^	0.471 ^b^	2.59 ^b^
Fructose	822 ^b^	3.00 ^a^	0.675	0.558 ^a^	22.9 ^b^	3.25 ^a^	0.681 ^a^	3.47 ^a^
*p value*	*0.048*	<*0.001*	*NS*	*0.018*	<*0.001*	<*0.001*	<*0.001*	<*0.001*
Interaction E × D								
*p value*	*NS*	*0.022*	*NS*	*0.046*	*0.045*	*NS*	*NS*	*0.008*

* C, control fed a diet with 65.8% corn starch; F, fed a diet with 65.0% fructose (F) added at the expense of corn starch; C + ME, fed a corn starch diet with a monomeric ET-rich extract; F + ME, fed a fructose diet with a monomeric ET-rich extract; C + DE, fed a corn starch diet with a dimeric ET-rich extract; F + DE, fed a fructose diet with a dimeric ET-rich extract. ^a,b,c,d^ Mean values within a column with different superscript letters were shown to be significantly different (*p *< 0.05); differences among the groups (C, F, C + ME, F + ME, C + DE, and F + DE) are indicated with superscripts only in the case of a statistically significant interaction E × D (*p *< 0.05). HDL, high-density lipoprotein; LDL, low-density lipoprotein; TC, total cholesterol; TG, triglycerides.

**Table 9:** Antioxidant status indices of rats fed experimental diets *.

	Serum		Liver							Heart		Kidneys	
	ACW	ACL	Mass ^1^	TBARS	TG	TC	GSH	GSSG	GSH/GSSG	Mass ^1^	TBARS	Mass ^1^	TBARS
	µg/mL	µg/mL		µg/g	µmol/g	µmol/g	µmol/g	µmol/g			µg/g		µg/g
Group *n* = 8													
C	3.01 ^d^	34.2	4.07	3.22	20.1 ^c^	22.0 ^d^	25.6	12.6 ^c^	2.09 ^a^	0.238	5.58	0.545 ^c^	6.64 ^c^
F	2.93 ^d^	33.7	4.94	4.37	45.1 ^a^	55.3 ^a^	30.0	24.1 ^a^	1.25 ^b^	0.249	6.54	0.693 ^a^	8.31 ^a^
C + ME	4.47 ^a^	37.0	4.11	3.46	20.6 ^c^	21.9 ^d^	23.0	12.7 ^c^	1.92 ^a^	0.249	5.83	0.570 ^c^	5.59 ^d^
F + ME	3.36 ^c,d^	38.3	4.75	3.80	33.4 ^b^	39.4 ^c^	27.2	15.8 ^c^	1.74 ^a,b^	0.250	6.19	0.633 ^b^	7.56 ^b^
C + DE	3.49 ^b,c^	34.3	4.03	3.53	21.5 ^c^	23.3 ^d^	33.0	15.9 ^c^	2.13 ^a^	0.247	5.80	0.560 ^c^	6.36 ^c^
F + DE	3.53 ^b^	36.5	4.84	3.97	37.9 ^a,b^	44.0 ^b^	29.3	19.9 ^b^	1.49 ^a,b^	0.253	6.44	0.649 ^b^	7.96 ^a,b^
*SEM*	*0.098*	*0.498*	*0.067*	*0.113*	*1.540*	*1.964*	*0.968*	*0.790*	*0.070*	*0.003*	*0.110*	*0.009*	*0.100*
Extract (E)													
- (without)	2.97 ^c^	33.9 ^b^	4.50	3.80	32.6 ^a^	38.7 ^a^	27.8 ^a,b^	18.4 ^a^	1.67	0.244	6.06	0.619	7.47 ^a^
ME	3.91 ^a^	37.7 ^a^	4.43	3.63	27.0 ^b^	30.7 ^b^	25.1 ^b^	14.2 ^b^	1.83	0.250	6.01	0.601	6.63 ^b^
DE	3.51 ^b^	35.4 ^b^	4.43	3.75	29.7 ^a,b^	33.6 ^b^	31.2 ^a^	17.9 ^a^	1.81	0.250	6.12	0.604	7.16 ^a,b^
*p value*	<*0.001*	*0.006*	*NS*	*NS*	*0.005*	<*0.001*	*0.029*	*0.007*	*NS*	*NS*	*NS*	*NS*	*0.030*
Diet (D)													
Corn starch	3.66 ^a^	35.2	4.07 ^b^	3.41 ^b^	20.7 ^b^	22.4 ^b^	27.2	13.7 ^b^	2.04 ^a^	0.245	5.74 ^b^	0.558 ^b^	6.23 ^b^
Fructose	3.27 ^b^	36.2	4.84 ^a^	4.05 ^a^	38.8 ^a^	46.2 ^a^	28.8	19.9 ^a^	1.49 ^b^	0.251	6.39 ^a^	0.658 ^a^	7.94 ^a^
*p value*	*0.007*	*NS*	<*0.001*	*0.004*	<*0.001*	<*0.001*	*NS*	<*0.001*	<*0.001*	*NS*	*0.003*	<*0.001*	<*0.001*
Interaction E × D													
*p value*	*0.002*	*NS*	*NS*	*NS*	*0.001*	<*0.001*	*NS*	*0.005*	*0.050*	*NS*	*NS*	*0.009*	*0.044*

* C, control fed a diet with 65.8% corn starch; F, fed a diet with 65.0% fructose (F) added at the expense of corn starch; C + ME, fed a corn starch diet with a monomeric ET-rich extract; F + ME, fed a fructose diet with a monomeric ET-rich extract; C + DE, fed a corn starch diet with a dimeric ET-rich extract; F + DE, fed a fructose diet with a dimeric ET-rich extract. ^a,b,c,d^ Mean values within a column with different superscript letters were shown to be significantly different (*p* < 0.05); differences among the groups (C, F, C + ME, F + ME, C + DE, and F + DE) are indicated with superscripts only in the case of a statistically significant interaction E × D (*p* < 0.05). ACL, integral antioxidant capacity of lipophilic substances in serum; ACW, integral antioxidant capacity of hydrophilic substances in serum; GSH, reduced glutathione; GSSG, oxidized glutathione; TBARS, thiobarbituric acid reactive substances; TC, total cholesterol; TG, triglycerides. ^1^ g/100 g body weight.

## Supplementary Materials

The following are available online at http://www.mdpi.com/2072-6643/10/4/445/s1, Table S1: Composition of the group-specific diets, Table S2: Large intestinal indices of rats fed experimental diets.

## References

[1] Basu A., Betts N.M., Nguyen A., Newman E.D., Fu D., Lyons T.J. (2014). Freeze-dried strawberries lower serum cholesterol and lipid peroxidation in adults with abdominal adiposity and elevated serum lipids. J. Nutr..

[2] Nile S.H., Park S.W. (2014). Edible berries: Bioactive components and their effect on human health. Nutrition.

[3] Forbes-Hernandez T.Y., Gasparrini M., Afrin S., Bompadre S., Mezzetti B., Quiles J.L., Giampieri F., Battino M. (2016). The healthy effects of strawberry polyphenols: Which strategy behind antioxidant capacity?. Crit. Rev. Food Sci. Nutr..

[4] Giampieri F., Tulipani S., Alvarez-Suarez J.M., Quiles J.L., Mezzetti B., Battino M. (2012). The strawberry: Composition, nutritional quality, and impact on human health. Nutrition.

[5] Cerda B., Tomas-Barberan F.A., Espín J.C. (2005). Metabolism of antioxidant and chemopreventive ellagitannis from strawberries raspberries, walnuts, and oak-aged wine in humans: Identification of biomarkers and individual variability. J. Agric. Food Chem..

[6] Aaby K., Mazur S., Nes A., Skrede G. (2012). Phenolic compounds in strawberry (*Fragaria* × *ananassa* Duch.) fruits: Composition in 27 cultivars and changes during ripening. Food Chem..

[7] Larrosa M., Garcia-Conesa M.T., Espín J.C., Tomas-Barberan F.A. (2010). Ellagitannins, ellagic acid and vascular health. Mol. Asp. Med..

[8] Landete J.M. (2011). Ellagitannins, ellagic acid and their derived metabolites: A review about source, metabolism, function and health. Food Res. Int..

[9] Garcia-Muñoz C., Vaillant F. (2014). Metabolic fate of ellagitannins: Implications for health, and research perspectives for innovative functional foods. Crit. Rev. Food Sci. Nutr..

[10] Żary-Sikorska E., Juśkiewicz J. (2008). Effects of fructans with different degrees of polymerization on bacterial enzymes activity, lipid profile and antioxidant status in rats. Pol. J. Food Nutr. Sci..

[11] Ghaffarzadegan T., Marungruang N., Nyman M. (2016). Molecular properties of guar gum and pectin modify cecal bile acids, microbiota, and plasma lipopolysaccharide-binding protein in rats. PLoS ONE.

[12] Saha P., Yeoh B.S., Singh R., Chandrasekar B., Vemula P.K., Haribabu B., Vijay-Kumar M., Jala V.R. (2016). Gut microbiota conversion of dietary ellagic acid into bioactive phytochemical urolithin A inhibits heme peroxidases. PLoS ONE.

[13] Juśkiewicz J., Jurgoński A., Kołodziejczyk K., Kosmala M., Milala J., Zduńczyk Z., Fotschki B., Żary-Sikorska E. (2016). Blood glucose lowering efficacy of strawberry extracts rich in ellagitannins with different degree of polymerization in rats. Pol. J. Food Nutr. Sci..

[14] Jurgoński A., Juśkiewicz J., Fotschki B., Kołodziejczyk K., Milala J., Kosmala M., Grzelak-Blaszczyk K., Markiewicz L. (2017). Metabolism of strawberry mono- and dimeric ellagitannins in rats fed a diet containing fructooligosaccharides. Eur. J. Nutr..

[15] Kosmala M., Zduńczyk Z., Juśkiewicz J., Jurgoński A., Karlińska E., Macierzyński J., Jańczak R., Rój E. (2015). Chemical composition of defatted strawberry and raspberry seeds and the effect of these dietary ingredients on polyphenol metabolites, intestinal function, and selected serum parameters in rats. J. Agric. Food Chem..

[16] Cerda B., Espín J.C., Parra S., Martinez P., Tomas-Barberan F.A. (2004). The potent in vitro antioxidant ellagitannins from pomegranate juice are metabolised into bioavailable but poor antioxidant hydroxy-6H-dibenzopyran-6-one derivatives by the colonic microflora of healthy humans. Eur. J. Nutr..

[17] Sójka M., Klimczak E., Macierzynski J., Kołodziejczyk K. (2013). Nutrient and polyphenolic composition of industrial strawberry press cake. Eur. Food Res. Technol..

[18] Kennedy J.A., Jones G.P. (2001). Analysis of proanthocyanidin cleavage products following acid-catalysis in the presence of excess phloroglucinol. J. Agric. Food Chem..

[19] Reeves P.G. (1997). Components of the AIN-93 diets as improvements in the AIN-76A diet. J. Nutr..

[20] Jurgoński A., Juśkiewicz J., Zduńczyk Z. (2013). An anthocyanin-rich extract from Kamchatka honeysuckle increases enzymatic activity within the gut and ameliorates abnormal lipid and glucose metabolism in rats. Nutrition.

[21] Zduńczyk Z., Juśkiewicz J., Wroblewska M., Krol B. (2004). Physiological effects of lactulose and inulin in the caecum of rats. Arch. Anim. Nutr..

[22] Botsoglou N.A., Fletouris D.J., Papageorgiou G.E., Vassilopoulos V.N., Mantis A.J., Trakatellis A.G. (1994). Rapid, sensitive, and specific thiobarbituric acid method for measuring lipid peroxidation in animal tissue, food, and feedstuff samples. J. Agric. Food Chem..

[23] Rahman I., Kode A., Biswas S.K. (2006). Assay for quantitative determination of glutathione and glutathione disulfide levels using enzymatic recycling method. Nat. Protoc..

[24] Folch J., Lees M., Sloane Stanley G.H. (1957). A simple method for the isolation and purification of total lipids from animal tissues. J. Biol. Chem..

[25] Gonzalez-Barrio R., Truchado P., Ito H., Espín J.C., Tomas-Barberan F.A. (2011). UV and MS identification of urolithins and nasutins, the bioavailable metabolites of ellagitannins and ellagic acid in different mammals. J. Agric. Food Chem..

[26] Fotschki B., Milala J., Jurgoński A., Karlinska E., Zduńczyk Z., Juśkiewicz J. (2014). Strawberry ellagitannins thwarted the positive effects of dietary fructooligosaccharides in rat cecum. J. Agric. Food Chem..

[27] Hansen M., Baunsgaard D., Autrup H., Vogel U.B., Moller P., Lindecrona R., Wallin H., Poulsen H.E., Loft S., Dragsted L.O. (2008). Sucrose, glucose and fructose have similar genotoxicity in the rat colon and affect the metabolome. Food Chem. Toxicol..

[28] Gugolek A., Juśkiewicz J., Strychalski J., Konstantynowicz M., Zwolinski C. (2015). Nutrient digestibility and colonic fermentation processes in species of the families *Mustelidae* and *Canidae* fed the same diet. J. Exp. Zool..

[29] Juśkiewicz J., Krol B., Kosmala M., Milala J., Zduńczyk Z., Żary-Sikorska E. (2015). Physiological properties of dietary ellagitannin-rich preparations obtained from strawberry pomace using different extraction methods. Pol. J. Food Nutr. Sci..

[30] Fotschki B., Juśkiewicz J., Jurgoński A., Kołodziejczyk K., Milala J., Kosmala M., Zduńczyk Z. (2016). Anthocyanins in strawberry polyphenolic extract enhance the beneficial effects of diets with fructooligosaccharides in the rat cecal environment. PLoS ONE.

[31] García-Villalba R., Vissenaekens H., Pitart J., Romo-Vaquero M., Espín J.C., Grootaert C., Selma M.V., Raes K., Smagghe G., Possemiers S. (2017). Gastrointestinal simulation model TWIN-SHIME shows differences between human urolithin-metabotypes in gut microbiota composition, pomegranate polyphenol metabolism, and transport along the intestinal tract. J. Agric. Food Chem..

[32] Cerda B., Llorach R., Ceron J.J., Espín J.C., Tomas-Barberan F.A. (2003). Evaluation of the bioavailability and metabolism in the rat of punicalagin, an antioxidant polyphenol from pomegranate juice. Eur. J. Nutr..

[33] Espín J.C., Larrosa M., Garcia-Conesa M.T., Tomas-Barberan F.A. (2013). Biological significance of urolithins, the gut microbial ellagic acid-derived metabolites: the evidence so far. Evid. Based Complement. Altern. Med..

[34] Manach C., Scalbert A., Morand C., Rémésy C., Jiménez L. (2004). Polyphenols: food sources and bioavailability. Am. J. Clin. Nutr..

[35] Basu A., Wilkinson M., Penugonda K., Simmons B., Betts N.M., Lyons T.J. (2009). Freeze-dried strawberry powder improves lipid profile and lipid peroxidation in women with metabolic syndrome: Baseline and post intervention effects. Nutr. J..

[36] Jarosławska J., Juśkiewicz J., Wroblewska M., Jurgoński A., Król B., Zduńczyk Z. (2011). Polyphenol-rich strawberry pomace reduces serum and liver lipids and alters gastrointestinal metabolite formation in fructose-fed rats. J. Nutr..

[37] Sanchez-Lozada L.G., Tapia E., Jimenez A., Bautista P., Cristobal M., Nepomuceno T., Soto V., Avila-Casado C., Nakagawa T., Johnson R.J. (2007). Fructose-induced metabolic syndrome is associated with glomerular hypertension and renal microvascular damage in rats. Am. J. Physiol. Renal Physiol..

[38] Kawasaki T., Igarashi K., Koeda T., Sugimoto K., Nakagawa K., Hayashi S., Yamaji R., Inui H., Fukusato T., Yamanouchi T. (2009). Rats fed fructose-enriched diets have characteristics of nonalcoholic hepatic steatosis. J. Nutr..

[39] Basciano H., Federico L., Adeli K. (2005). Fructose, insulin resistance, and metabolic dyslipidemia. Nutr. Metab..

[40] Jarosławska J., Wróblewska M., Juśkiewicz J., Brzuzan L., Zduńczyk Z. (2016). Protective effects of polyphenol-rich blackcurrant preparation on biochemical and metabolic biomarkers of rats fed a diet high in fructose. J. Anim. Physiol. Anim. Nutr..

[41] Mazzone G., Toscano M., Russo N. (2013). Density functional predictions of antioxidant activity and UV spectral features of nasutin A, isonasutin, ellagic acid, and one of its possible derivatives. J. Agric. Food Chem..

[42] Rieckmann P., Tuscano J.M., Kehrl J.H. (1997). Tumor necrosis factor-α (TNF-α) and interleukin-6 (IL-6) in B-lymphocyte function. Methods.

[43] Tseng W.P., Tsu C.M., Tang C.H. (2010). FAK activation is required for TNF-α induced IL-6 production in myoblasts. J. Cell. Physiol..

