# Supplementary material for: Protective Effects of Ellagitannin-Rich Strawberry Extracts on Biochemical and Metabolic Disturbances in Rats Fed a Diet High in Fructose
Source: Nutrients. 2018 Apr 4;10(4):445. doi: 10.3390/nu10040445 (PMC5946230; doi:10.3390/nu10040445)
Supplement: Supplementary file 1 [file nutrients-10-00445-s001.pdf]

nutrients

MDPI

Protective Effects of Ellagitannin-Rich Strawberry Extracts on Biochemical and Metabolic Disturbances in Rats Fed a Diet High in Fructose

Bartosz Fotschki [1,*], **Jerzy** Juśkiewicz [1,*], **Krzysztof** Kołodziejczyk [2], **Adam** Jurgoński [1], **Monika** Kosmala [2], **Joanna** Milala [2], **Katarzyna** Ognik [3] **and Zenon** Zduńczyk [1]

[1] Institute of Animal Reproduction and Food Research, Division of Food Science, Tuwima 10, 10-748 Olsztyn, Poland; a.jurgonski@pan.olsztyn.pl (A.J.); z.zdunczyk@pan.olsztyn.pl (Z.Z.)

[2] Institute of Food Technology and Analysis, Lodz University of Technology, Stefanowskiego 4/10, 90-924 Lodz, Poland; krzysztof.kolodziejczyk@p.lodz.pl (K.K.); monika.kosmala@p.lodz.pl (M.K.); joanna.milala@p.lodz.pl (J.M.)

[3] Department of Biochemistry and Toxicology, Faculty of Biology, Animal Sciences and Bioeconomy, University of Life Sciences, 20-950 Lublin, Poland; kasiaognik@poczta.fm

* Correspondence: b.fotschki@pan.olsztyn.pl (B.F.); j.juskiewicz@pan.olsztyn.pl (J.J.); Tel.: +48-89-523-46-01 (B.F.); +48-89-523-46-73 (J.J.)

Supplementary materials:

Table S1. Composition of the group-specific diets.

	Group (%)					
	C	F	C+ME	F+ME	C+DE	F+DE
Casein	14.8	14.8	14.8	14.8	14.8	14.8
Cellulose[1]	6	6	6	6	6	6
Rapeseed oil	8	8	8	8	8	8
Mineral mix[2]	3.5	3.5	3.5	3.5	3.5	3.5
Vitamin mix[3]	1	1	1	1	1	1
Choline chloride	0.2	0.2	0.2	0.2	0.2	0.2
DL-methionine	0.2	0.2	0.2	0.2	0.2	0.2
Cholesterol	0.5	0.5	0.5	0.5	0.5	0.5
Monomeric ET-rich extract	0	0	0.23	0.23	0	0
Dimeric ET-rich extract	0	0	0	0	0.24	0.24
Corn starch	65.8	0.8	65.57	0.57	65.56	0.56
Fructose	0	65.0	0	65.0	0	65.0

Calculated dietary contents						
Total polyphenols	0	0	0.203	0.203	0.197	0.197
Ellagitannins *(monomer:dimer ratio)*	0	0	0.184 *(96:4)*	0.184 *(96:4)*	0.138 *(40:60)*	0.138 *(40:60)*
Proanthocyanidins	0	0	0.019	0.019	0.057	0.057

ET – ellagitannins.

[1] The α-cellulose preparation was obtained from Sigma-Aldrich (No. C8002).

[2] AIN-93G (Reeves 1997)[18], g per kg mix: 357 g anhydrous calcium carbonate (40.04% Ca), 196 g monobasic potassium phosphate (22.76% P, 28.73% K), 70.78 g potassium citrate and tripotassium monohydrate (36.16% K), 74 g sodium chloride (39.34% Na, 60.66% Cl), 46.6 g potassium sulfate (44.87% K, 18.39% S), 24 g magnesium oxide (60.32% Mg), 6.06 g ferric citrate (16.5% Fe), 1.65 g zinc carbonate (52.14% Zn), 1.45 g sodium meta-silicate 9 $9H_2O$ (9.88% Si), 0.63 g manganous carbonate (47.79% Mn), 0.3 g cupric carbonate (57.47% Cu), 221.026 g powdered sucrose, and 0.275 g chromium potassium sulfate × $12H_2O$ (10.42% Cr). The following components were added in mg per kg mix quantities: 81.5 mg boric acid (17.5% B), 63.5 mg sodium fluoride (45.24% F), 31.8 mg nickel carbonate (45% Ni), 17.4 mg lithium chloride (16.38% Li), 10.25 mg anhydrous sodium selenate (41.79% Se), 10 mg potassium iodate (59.3% I), 7.95 mg ammonium paramolybdate × $4H_2O$ (54.34% Mo), and 6.6 mg ammonium vanadate (43.55% V).

[3] AIN-93G (Reeves 1997)[18], g per kg mix: 3.0 g nicotinic acid, 1.6 g Ca pantothenate, 0.7 g pyridoxine–HCl, 0.6 g thiamine-HCl, 974.655 g powdered sucrose, 0.6 g riboflavin, 0.2 g folic acid, 0.02 g biotin, 2.5 g vit. B_{12} (cyanocobalamin, 0.1% in mannitol). The following components were added in IU per g quantities: 15.0 IU vit. E (all-rac-α-tocopheryl acetate, 500), 0.8 IU vit. A (all-trans-retinyl palmitate, 500000), 0.25 IU vit. D_3 (chole-calciferol, 400000), and 0.075 IU vit. K-1 (phylloquinone).

Table S2. Large intestinal indices of rats fed experimental diets *.

	Cecum					Colon		
	tissue[1]	digesta[1]	DM, %	NH_3, mg/g	pH	tissue[1]	digesta[1]	pH
Group (n=8)								
C	0.162	0.512	24.7	0.234	7.38	0.296	0.313	7.65
F	0.170	0.661	25.3	0.247	7.44	0.337	0.504	9.64
C+ME	0.168	0.529	25.3	0.240	7.36	0.287	0.324	7.62
F+ME	0.167	0.504	25.7	0.241	7.40	0.326	0.383	7.57
C+DE	0.162	0.529	25.6	0.257	7.46	0.294	0.294	7.66
F+DE	0.178	0.599	26.5	0.231	7.45	0.295	0.393	7.66
SEM	*0.002*	*0.017*	*0.302*	*0.006*	*0.035*	*0.008*	*0.021*	*0.038*
Extract (E)								
- (without)	0.166	0.586	25.0	0.241	7.41	0.316	0.408	7.64
ME	0.167	0.516	25.5	0.240	7.38	0.307	0.353	7.59

DE	0.170	0.564	26.1	0.244	7.46	0.294	0.344	7.66
P value	*NS*	*NS*	*NS*	*NS*	*NS*	*NS*	*NS*	*NS*
Diet (D)								
Corn starch	0.164	0.523[b]	25.2	0.244	7.40	0.292	0.310[b]	7.64
Fructose	0.172	0.588[a]	25.8	0.240	7.43	0.319	0.427[a]	7.62
P value	*NS*	*0.046*	*NS*	*NS*	*NS*	*NS*	*0.004*	*NS*
Interaction E×D								
P value	*NS*	*NS*	*NS*	*NS*	*NS*	*NS*	*NS*	*NS*

* C, control fed a diet with 65.8% corn starch; F, fed a diet with 65.0% fructose (F) added at the expense of corn starch; C+ME, fed a corn starch diet with a monomeric ET-rich extract; F+ME, fed a fructose diet with a monomeric ET-rich extract; C+DE, fed a corn starch diet with a dimeric ET-rich extract; F+DE, fed a fructose diet with a dimeric ET-rich extract. [a,b] Mean values within a column with unlike superscript letters were shown to be significantly different ($P<0.05$); differences among the groups (C, F, C+ME, F+ME, C+DE, F+DE) are indicated with superscripts only in the case of a statistically significant interaction E×D ($P<0.05$). [1] mass, g/100 g BW; DM, dry matter.
